# 235 Impact of Plasma Next Generation Sequencing (pNGS) on CLABSI Rates

**DOI:** 10.1017/ash.2026.10611

**Published:** 2026-06-23

**Authors:** Jenna Rasmusson, Priya Sampathkumar, Nancy Wengenack, Michelle Meyer, Alyssa Olson

**Affiliations:** 1 Mayo Clinic; 2 Mayo Clinic, Rochester, MN

## Abstract

**Background:** Candida auris is a globally emerging, multidrug-resistant fungal pathogen that causes serious, difficult-to-treat infections in hospitalized patients. C. auris cases in the United States have been linked to receipt of healthcare overseas. Outbreaks have also occurred in New York City, New Jersey, Illinois, Nevada, and California. We provide care to patients from all 50 states and 138 countries and are therefore, at risk for encountering C.auris within our facility. **Methods:** A case-finding tool was created by Infection Prevention and Control (IPAC) within the electronic medical record (EMR) to identify patients for screening. Inclusion criteria was centric to patients who were admitted in the previous 24 hours with a primary address in a foreign country or in areas of the United States with known C. auris transmission. Patients who are positive for Carbapenamase-producing organisms (CPO) are also included in screening criteria. In 2020, Clinical Microbiology validated a polymerase chain reaction (PCR) test in partnership with the State Health Department and IPAC. In January 2022, IPAC created a protocol that allows IPAC staff to order C. auris screening directly in the EMR utilizing the validated PCR test. The order is paired with communication from IPAC alerting the patient care team that C.auris screening is indicated. Instructions on test collection and information on C. auris are also included in messaging. The patient’s primary nurse then obtains verbal consent from the patient to collect a composite axilla–groin skin swab. Swabs are sent to Clinical Microbiology for testing, and results are reported directly into the EMR. **Results:** Since the introduction of the nursing protocol in January 2022, 2,660 patients have been identified for C. auris screening through November 2025. Of the 2,660 patients identified for testing, 1,111 patients had screening completed. Two patients tested positive for C. auris on screening. Both patients had prolonged travel to regions with known C. auris transmission. **Conclusion:** The EMR can be leveraged for early identification and screening of patients at risk of C.auris colonization. Case finding tools, combined with nursing protocols and communication strategies within the EMR can be effectively replicated and modified to respond to emerging infections.

